# Wumei Pill Ameliorates AOM/DSS-Induced Colitis-Associated Colon Cancer through Inhibition of Inflammation and Oxidative Stress by Regulating S-Adenosylhomocysteine Hydrolase- (AHCY-) Mediated Hedgehog Signaling in Mice

**DOI:** 10.1155/2022/4061713

**Published:** 2022-07-26

**Authors:** Jue Wang, Kang Ding, Yuhang Wang, Tingdong Yan, Yun Xu, Zirong Deng, Weiling Lin, Libei Zhang, Weizhong Zhu, Rui Zhao, Yuhang Zhou, Zhaoguo Liu

**Affiliations:** ^1^School of Pharmacy, Nantong University, 19 Qixiu Road, Nantong, Jiangsu Province 226001, China; ^2^National Center of Colorectal Surgery, Jiangsu Integrate Colorectal Oncology Center, Nanjing Hospital of Chinese Medicine Affiliated to Nanjing University of Chinese Medicine, Nanjing 210022, China

## Abstract

Wumei Pill (WMP) is a traditional Chinese herbal formulation and widely used to treat digestive system diseases in clinical. S-Adenosylhomocysteine hydrolase (AHCY) can catalyze the hydrolysis of S-adenosylhomocysteine to adenosine and homocysteine in living organisms, and its abnormal expression is linked to the pathogenesis of many diseases including colorectal cancer (CRC). A previous study reported that WMP could prevent CRC in mice; however, the underlying mechanisms especially the roles of AHCY in WMP-induced anti-CRC remain largely unknown. Here, we investigated the regulatory roles and potential mechanisms of AHCY in WMP-induced anti-CRC. WMP notably alleviated the azoxymethane/dextran sulfate sodium- (AOM/DSS-) induced colitis-associated colon cancer (CAC) in mice. Besides, WMP inhibited the inflammation and oxidative stress in AOM/DSS-induced CAC mice. AHCY was high expression in clinical samples of colon cancer compared to the adjacent tissues. WMP inhibited the AHCY expression in AOM/DSS-induced CAC mice. An *in vitro* study found that AHCY overexpression induced cell proliferation, colony formation, invasion, and tumor angiogenesis, whereas its knockdown impaired its oncogenic function. AHCY overexpression enhanced, while its knockdown weakened the inflammation and oxidative stress in colon cancer cells. Interestingly, WMP potently suppressed the hedgehog (Hh) signaling in AOM/DSS-induced CAC mice. A further study showed that AHCY overexpression activated the Hh signaling while AHCY knockdown inactivated the Hh signaling. Moreover, activation of the Hh signaling reversed the effect of AHCY silencing on inflammation and oxidative stress *in vitro*. In conclusion, WMP alleviated the AOM/DSS-induced CAC through inhibition of inflammation and oxidative stress by regulating AHCY-mediated hedgehog signaling in mice. These findings uncovered a potential molecular mechanism underlying the anti-CAC effect of WMP and suggested WMP as a promising therapeutic candidate for CRC.

## 1. Introduction

Colorectal cancer (CRC) is the third most frequently diagnosed cancer and the fourth leading cause of cancer-related death globally [1]. The risk factors for CRC include age, gender, obesity, excessive alcohol consumption, smoking, red and processed meals, family history of colorectal cancer, and inflammatory bowel diseases (IBD) [2]. CRC is prone to distant metastases to other organs, and liver metastasis is the main cause of colon cancer-related death [3]. The current treatment approaches to CRC include surgical resection combined with radio- and chemotherapy [4]. However, adverse effects and drug resistance caused by radio- and chemotherapy have affected the efficacy and adherence to treatment and make the current treatments temporary and incomplete [5]. Although checkpoint-based immunotherapy has shown exciting results in the treatment of tumors, however, it has been found to be unresponsive to most solid tumors including CRC [6]. Traditional Chinese herbal formulation has been widely used to treat digestive system diseases in clinical practice in China for centuries and has achieved good therapeutic effect in treating CRC in preclinical and clinical trials [7–10]. However, the underlying mechanisms of those formulations remain elusive, which hinders its wide application in clinic. Therefore, it is particularly urgent to clarify its potential mechanisms.

Accumulating evidences have proven that chronic intestinal inflammation is closely linked to the pathogenesis of CRC [11, 12]. Inflammatory bowel diseases (IBD), including ulcerative colitis (UC) and Crohn's disease (CD), are chronic inflammatory disorder of the intestine and can result in a well-recognized increased risk of colon carcinogenesis [13, 14], especially for the development and progression of colitis-associated colorectal cancer (CAC) [15]. Upon the initiation of CAC, a large number of inflammatory cells, such as macrophage, natural killer cells, and dendritic cells, infiltrate around the colonic tissues [16, 17], leading to the increased production of proinflammatory cytokines, which makes colonic tissues in an inflammatory microenvironment. Without effective treatment, persistent inflammatory stimulation will further aggravate CAC [18]. Therefore, control of inflammation is pivotal for the prevention of CAC. Indeed, the increased proinflammatory cytokines could also promote the accumulation of reactive oxygen species (ROS) [19]. The accumulated ROS will further cause oxidative DNA damage, impair DNA repair and genomic instability, and finally trigger the colon tumorigenesis [20]. Besides, studies have reported that suppression of oxidative stress helped to control the progression of CAC [21], highlighting that control of oxidative stress is an effective strategy for combating CAC.

S-Adenosylhomocysteine hydrolase (AHCY) is one of the most conserved proteins in living organisms [22]. AHCY is the only mammalian enzyme known to mediate the reversible catalysis of S-adenosyl-L-homocysteine (SAH) to adenosine (Ado) and homocysteine (Hyc) [23]. Studies indicated that AHCY was essential for embryonic development and cellular stress, and proper activity of AHCY was required for keeping the cellular methylation potential [24]. The dysfunctional or abnormal expression of AHCY links to several pathological consequences, such as ischemic stroke [20], hepatitis B-induced liver cirrhosis [25], and age-related diseases and Alzheimer's disease [26]. In addition, stable knockdown experiments of AHCY have revealed its critical role in cancers [27, 28]. AHCY was initially identified as a tumor suppressor, and the mRNA expression of AHCY was found reduced in many solid cancer tissues. Interestingly, such function as a tumor suppressor was found cell type specific in later studies, as a study found that inhibition of AHCY could also result in anti-invasion and antimigration effects in breast cancer [28], and reduced AHCY activity could lead to cell cycle arrest and decreased proliferation in liver cancer cells [29]. Besides, AHCY inhibitor 3-deazaadenosine, a general methylation inhibitor that depletion of S-adenosylmethionine, was proved to have anti-inflammatory properties in murine and human macrophages [30]. However, the roles and regulatory effects of AHCY in CRC remain largely unknown.

Hedgehog (Hh) signaling is a highly conserved evolutionary pathway that controls complex developmental processed and cell homeostasis in vertebrates [31, 32]. The aberrant activation of Hh signaling links to several cancer types including CRC [33]. Compelling evidence indicates that Hh signaling plays essential roles in the organogenesis of the intestine and in adult intestine homeostasis [34] and is strongly implicated in the pathogenesis of intestinal inflammation [35]. Disruption of Hh signaling was found to participate in the development of IBD and has been considered a therapeutic target for IBD [36]. Furthermore, accumulating evidence showed that Hh signaling is involved in the progression of CAC, and inhibition of Hh signaling attenuated the CAC [37]. Besides, Hh signaling inhibitors showed good effects in preventing CAC by ameliorating oncogenic inflammation and suppressing tumor proliferation [38]. Therefore, searching inhibitors or modulators of Hh signaling are believed to be useful in the clinical application of CAC treatment.

Chinese herbal medicine has long been used to CRC treatment due to its high efficacy, safety, and relatively low economic costs [39]. Wumei Pill (WMP) is a classic prescription recorded in “Treatise on Febrile Disease”. As a traditional Chinese medicine (TCM), WMP is widely used to treat digestive system diseases in clinical practice in China [40]. Previous studies showed that WMP had extensive pharmacological effects, including alleviated trinitrobenzenesulfonic acid- (TNBS-) induced colitis [41], prevented high-fat diet-induced obesity [42], protected pancreatic *β* cells [43], and reduced insulin resistance [44]. Noteworthy, WMP can treat roundworm coldness in the extremities and longtime diarrhea of cold-heat complex type based on the “Treatise on Febrile Disease.” Although there is no record of CAC in TCM literatures, however, according to its clinical characteristics and pathogenesis, CAC can be classified as a category of “dysentery” and “risceral intoxication.” WMP is a famous formula, which is composed of ten herbs: *Fructus Mume*, *Herba Asari*, *Rhizoma Zingiberis*, *Rhizoma Coptidis*, *Radix Angelicae sinensis*, *Rhizoma Typhonii gigantei*, *Pericarpium Zanthoxyli*, *Ramulus cinnamomi*, *Radix Ginseng*, and *Cortex Phellodendri* [41]. Many preclinical and clinical trials found that WMP was suitable for the early prevention and treatment of inflammatory bowel disease and CAC. Interestingly, one study reported that WMP attenuated CAC by regulating the balance between “tumor-promoting bacteria” and “tumor-suppressing bacteria” and the NF-*κ*B/IL-6/STAT3 pathway in mice [45], highlighting that WMP was a potential effective chemo-preventive drug. However, in order to further promote the wide application of WMP in treatment of CAC, further clinical evidence and in-depth mechanism research are still needed. In this study, we hypothesized that modulation of AHCY-mediated Hh signaling contributed to the anti-inflammatory and antioxidative stress effects of WMP in AOM/DSS-induced CAC. To test the hypothesis, we first established an AOM/DSS-induced CAC model in mice and evaluated the anti-CAC, anti-inflammatory, and anti-oxidative stress effects of WMP *in vivo*. Then the effect of WMP on AHCY in CAC mice and the biological function of AHCY in colon cancer cells were investigated, including its regulatory roles in tumor cell proliferation, invasion, colony formation, tumor angiogenesis, inflammation, and oxidative stress in colon cancer cells. Then, the effect of WMP on Hh signaling in CAC mice and the regulatory effect of AHCY on inflammation and oxidative stress in colon cancer cells were assessed. Finally, the roles of Hh signaling in AHCY-mediated regulation of inflammation and oxidative stress were further clarified.

## 2. Materials and Methods

### 2.1. Chemicals and Reagents

Wumei Pill (3 g/pill, Lot No. 20200815) was purchased from Yunnan Tengyao Pharmaceutical Co., Ltd. AOM (A5486) was purchased from Sigma. DSS (Cat No. 160110) was purchased from MP Biomedicals, LLC. L-OHP (2205104) was purchased from Jiangsu Hengrui Pharmaceuticals Co., Ltd. SW480, Colo-205, RKO, Caco-2, DLD-1, HCT116, HCT15, SW620, and LOVO cells were purchased from Shanghai Zhong Qiao Xin Zhou Biotechnology Co., Ltd. Primary antibody against AHCY (SAB1405439) was purchased from Sigma. Primary antibodies against Patched (ab53715), Gli1 (ab217326), COX-2 (ab15191), and iNOS (ab178945) were purchased from Abcam. Primary antibodies against Smo (sc-166685) and Hhip (sc-293265) were purchased from Santa Cruz. Primary antibody against Lamin B1 (AF1408) was purchased from Beyotime. Shh ELISA kit (MM-1130M2) was purchased from MEIMIAN. SAG (SF6836) was purchased from Beyotime. ELISA kits including murine IL-1*β* kit (RAB0275) and murine IL-6 kit (RAB0308) were purchased from Sigma. ELISA kits including human interleukin-1*β* (IL-1*β*) kit (PI305) and human IL-6 kit (PI330) were purchased from Beyotime. Detection kits including MDA (A003-4-1), GSH (A006-2-1), GSH-Px (A005-1), and SOD (A001-3) were purchased from Nanjing Jiancheng Bioengineering Institute. CAT (ab83464) detection kit was purchased from Abcam. CCK8 (C0043) kit was purchased from Beyotime. Matrixgel (#356234) was purchased from Corning incorporated. AHCY shRNA and AHCY pcDNA3.1(+) plasmids were purchased from Suzhou GenePharma Co., Ltd. Lipofectamine 2000 (MAN0007824) was purchased from Invitrogen.

### 2.2. Patients and Tissue Specimens

From 2019 to 2021, 68 pairs of fresh CRC tissues and paired normal colorectal tissues from patients with primary CRC were collected from the Nanjing Hospital of Chinese Medicine. All CRC tissues were diagnosed histologically, and none of these patients received any chemotherapy or radiotherapy before the operation. This study was permitted and approved by the Ethics Committee of Nanjing Hospital of Chinese Medicine (KY2020034).

### 2.3. WMP Preparation

Wumei Pill (Lot No.20200815) was purchased from Yunnan Tengyao Pharmaceutical Co., Ltd. As a marked drug, the weight of the pill is 3 g/pill. To obtain the indicated dosages, the detailed preparation process was as follows: For WMP (3 g/kg) preparation, take 6 g pill, then cut and ground the pills, add an appropriate amount of 0.5% CMC-Na to suspend the solution (volume of 20 mL); WMP was given to mice by gastric gavage in 0.1 mL/10 g body weight. For WMP (6 g/kg) preparation, take 12 g pill, then cut and ground the pills, add an appropriate amount of 0.5% CMC-Na to suspend the solution (volume of 20 mL); WMP was given to mice by gastric gavage in 0.1 mL/10 g body weight.

### 2.4. Animals and Experimental Design

Male C57BL/6 mice (*n* = 90, six weeks old, 20 ± 2 g) were provided by Experimental Animal Center of Nantong University (Nantong, China). The study was approved by the Institutional Animal Ethical Committee of Nantong University (approval No. S20200907-306). The experimental procedures were conducted according to NIH Guidelines for Care and Use of Laboratory Animals. Mice were kept in a room under a 12/12 h day/night cycle. All mice were given free access to diet and water during the course of experiments. Mice were allowed to adapt to the Experimental Animal Laboratory for 1 week before the commencement of experiment. Mice were randomly divided into five groups, i.e., the control group, AOM/DSS group, WMP (3 g/kg) group, WMP (6 g/kg) group, and L-OHP (5 mg/kg) group. The control group has 10 mice; other groups have 20 mice per group. Oxaliplatin (L-OHP) was treated as a positive drug. To establish the CAC model, mice received an intraperitoneal injection of AOM (10 mg/kg) dissolved in physiological saline. After 7 days, the animals were provided with drinking water containing 2.5% DSS for 7 days, followed by drinking water for 14 days, and exposed to two more 2.5% (*w*/*v*) DSS treatment cycles. WMP (3 g/kg) and WMP (6 g/kg) were given to mice by gastric gavage once a day for four weeks (week 9 to 12, day 57 to 84) after suspending DSS treatment (week 8, day 56). L-OHP was given to mice by intraperitoneal injection once a week for four weeks (week 9 to 12, day 57 to day 84) after suspending DSS treatment (week 8, day 56). The control group was given equivalent normal saline. The mice were weighed weekly and sacrificed at the end of week 12 (day 84). The colons of mice were removed for length measurement. After colon length measurement, the colons were cut longitudinally and then washed with PBS (phosphate-buffered saline, pH 7.4, 4°C) for macroscopical inspection. The number of tumors in the colons was recorded based on gross examination. After recording tumor numbers, the colons were cut into 1 cm pieces and then fixed in 10% buffered formalin (pH 7.4). Remaining samples were flash-frozen in liquid nitrogen and stored at -80°C for immunohistochemistry and western blot protein analysis.

### 2.5. Histopathological Examination

The segregated colon samples were fixed instantly with 10% (*v*/*v*) formalin and embedded. The paraffin-embedded samples were sectioned to 5 *μ*m thickness and stained with hematoxylin-eosin staining (H&E) for histological analysis. The detailed process was reported previously [46]. Representative images were measured under the microscope.

### 2.6. Immunohistochemical Staining

Paraffin-embedded tissues were used for analyzing the expression of AHCY according to previously reported [47]. Briefly, tissue sections were deparaffinized and rehydrated using a graded ethanol series and distilled water and then treated with 3% H_2_O_2_ in methanol for 30 min then washed with PBS; 10% normal goat serum was incubated for 30 min. After washing, primary antibody AHCY (1 : 500) and proliferating cell nuclear antigen (PCNA) (1 : 300) were applied to tissue overnight at 4°C. Sections were then washed in PBS three times and incubated with secondary antibodies. Following washing, sections were developed with DAB using a commercial kit (CoWin Biosciences), counterstained with hematoxylin, and cover slipped. Finally, observation and taking photos were performed under a standing microscope.

### 2.7. Disease Activity Index (DAI)

During the DSS cycle, the clinical manifestations of mice were monitored and recorded using the disease activity index (DAI) as previously reported [48]. Briefly, DAI was calculated as the sum score according to the following indicators: body weight loss (0 points, none; 1 point, <5%; 2 points, 5%-10%; 3 points: 10%-20%; 4 points, >20%); diarrhea (0 points, normal; 2 points, loose stools; 4 points, watery diarrhea); and rectal bleeding (0 points, no bleeding; 2 points, slight bleeding; 4 points, gross bleeding).

### 2.8. Western Blot Analyses

Proteins from colon tissues and colon cancer cells were extracted by RIPA lysis buffer supplemented with 1% of PMSF and protease inhibitor cocktail followed the standard protocol. Protein concentration was determined by bicinchoninic acid (BCA) protein assay kit. The protocol of Western blot analysis was according to previously reported [49]. Target proteins, such as iNOS, COX-2, AHCY, Patched, Smo, Hhip, and Gli 1, were detected by corresponding primary antibodies and subsequently by horseradish peroxidase-conjugated secondary antibodies. Equivalent loading was confirmed using antibodies against GAPDH or Lamin B1. Densitometry analysis was performed using the ImageJ software. Representative blots were from three independent experiments.

### 2.9. Enzyme-Linked Immunosorbent Assay (ELISA)

IL-1*β*, IL-6, and Shh concentrations were determined in the plasma of CAC mice or in the supernatants of colon cancer cells according to the manufacturer's instructions [50]. Results were from three independent experiments.

### 2.10. Plasmid Transfection

AHCY shRNA, the negative control shRNA, AHCY pcDNA3.1(+) plasmid, and the negative control vector were transfected into SW620 or DLD-1 cells using the lipofectamine 2000 transfection reagent according to the manufacturer's instructions [51]. The transfection efficiency was confirmed by western blot analysis. Results were from three independent experiments.

### 2.11. CCK8 Assay

The cell counting kit-8 (CCK-8) assay was used as a qualitative index of cell proliferation. 1 × 10^4^ cells were plated in 96-well microplates, and cell counts were performed using a CCK-8 assay according to the manufacturer's protocol. Briefly, 10 *μ*L of CCK-8 solution was added to each well, and the samples were incubated for 1 h before the absorbance was measured at 450 nm. Results were from three independent experiments.

### 2.12. Transwell Invasion Assay

Transwell experiment was used to detect cell invasion ability as reported previously [52]. Briefly, matrixgel was spread onto the upper chamber. 2 × 10^5^ cells (transfection with AHCY shRNA or AHCY pcDNA3.1(+) plasmid) were seeded into the chamber and cultured in a serum-free medium. Then, Dulbecco's modified Eagle's medium (DMEM) medium containing 10% FBS was added to the lower chamber. After culturing 20 h in an incubator containing 5% CO_2_ at 37°C, the medium was removed. The invasion cells were stained with 0.1% crystal violet for 15 min and then washed with running water. The invasion cells were observed under a microscope. Results were from three independent experiments.

### 2.13. Capillary Tube Formation Assay

Capillary tube formation assay was conducted as reported by our previous publication [53]. Briefly, matrigel were added in 96-well plates (100 *μ*L/well) and placed in incubator for 30 min at 37°C until solidification; then primary human umbilical vein endothelial cells (HUVECs) in serum-free media were seeded in 96-well plates (1 × 10^4^ cells/well) with SW620 or DLD-1 cultural supernatant. After a 12 h incubation of HUVEC cells with SW620 or DLD-1 cultural supernatant, capillary tube formation was quantified. Five fields were imaged (×100 magnification) with an inverted microscope equipped with a digital camera and the degree of tubulogenesis was quantified by counting branch points. Results were from three independent experiments.

### 2.14. Biochemical Estimations

Levels of malondialdehyde (MDA) and glutathione (GSH) and enzymatic activities of superoxide dismutase (SOD), catalase (CAT), and glutathione peroxidase (GSH-Px) in the colon tissues of CAC mice or in the colon cancer cells were detected by using detection kits according to the protocols, respectively. Results were from three independent experiments.

### 2.15. Statistical Analysis

All data were expressed as percentage and mean ± SD. Statistical analysis was performed using Student's *t*-test and one-way ANOVA by GraphPad Prism 5 for Windows. Values of *p* < 0.05 were considered to be statistically significant.

## 3. Results

### 3.1. WMP Ameliorated the AOM/DSS-Induced Colitis-Associated Colon Cancer (CAC) in Mice

**Figure 1:**
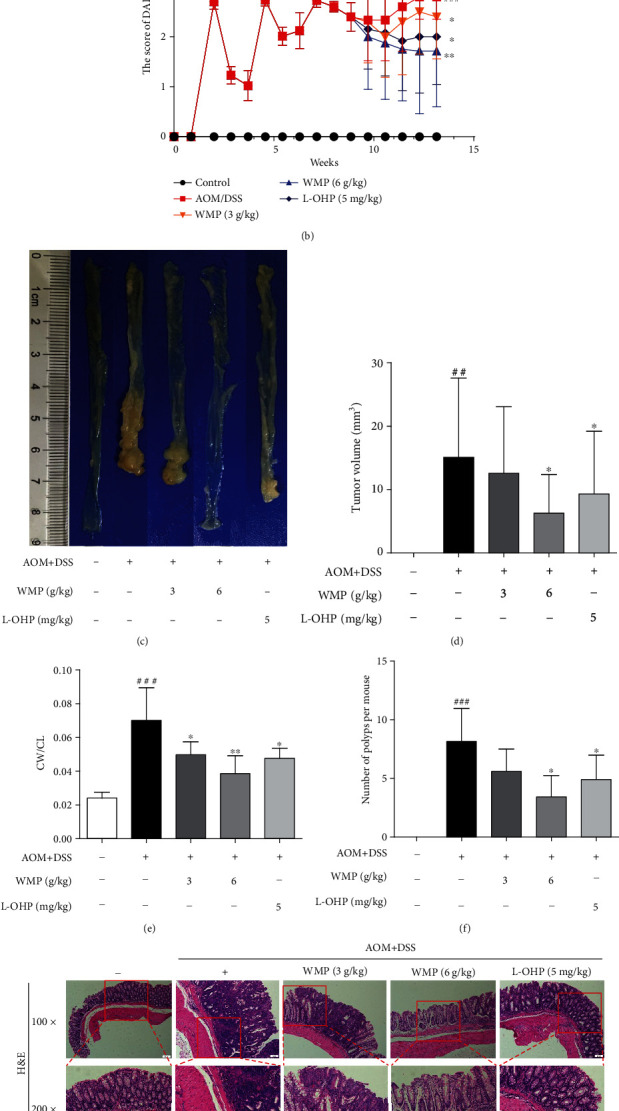
WMP ameliorated the AOM/DSS-induced colitis-associated colon cancer (CAC) in mice. (a) Body weight change was recorded every week. (b) The score of disease activity index (DAI). (c) Representative gross morphology of mice colon tissues. (d) Tumor volume. (e) The ratio of colon weight (CW)/colon length (CL) of mice. (f) The total number of colon tumors. (g) Representative pictures of H&E staining were shown. (h) Immunohistochemical analyses of PCNA protein levels in the colon tissues of mice. Data are expressed as the mean ± SD of 7-10 mice in each group. ^##^*p* < 0.01 and^###^*p* < 0.001 versus the control group; ^∗^*p* < 0.05 and^∗∗^*p* < 0.01 versus the AOM/DSS group.

A previous study has proved that WMP could attenuate CAC [45]; however, the previous study has few detection indicators and especially lacks the positive control drug and H&E pathological examination. Therefore, to determine the therapeutic effect of WMP on CRC, we also established the AOM/DSS-induced colitis-associated colon cancer (CAC) model in mice. Oxaliplatin (L-OHP) was selected as the positive control drug in our study. AOM/DSS stimulation decreased the body weight and increased the DAI score of mice compared to the control group; however, they were reversed by treatment with WMP (Figures 1(a) and 1(b)). Compared to the control group, macroscopic tumors and shortened colon length were observed in mice given AOM/DSS stimulation. Besides, compared to the control group, AOM/DSS stimulation also increased the number of polyps per mouse, the tumor volume, and the ratio of colon weight (CW)/colon length (CL) of mice (Figures 1(c)–1(f)). However, treatment with WMP in mice restored the colon length (Figure 1(c)) and decreased the number of polyps per mouse, the tumor volume, and CW/CL (Figures 1(d)–1(f)). L-OHP also ameliorated the AOM/DSS-induced changes mentioned above similar to WMP, but the effect was slightly lower than that of the high dosage (6 g/kg) of the WMP group (Figures 1(c)–1(f)). H&E staining was used to evaluate the effect of WMP on the pathological changes caused by AOM/DSS stimulation. Pathological examination showed that AOM/DSS stimulation led to the destruction of intestinal epithelial structure, large area edema, and massive infiltration of inflammatory cells and was evidently ameliorated by administration with WMP and L-OHP (Figure 1(g)). The expression level of proliferating cell nuclear antigen (PCNA) was further examined by immunohistochemistry staining. Results showed that PCNA was highly expressed in the AOM/DSS-treated group compared to the control group, and treatment with WMP and L-OHP lowered the PCNA expression (Figure 1(h)). Taken together, the aggregated results suggested that WMP ameliorated the AOM/DSS-induced CAC in mice.

### 3.2. WMP Inhibited Inflammation and Oxidative Stress in Colon Tissues of AOM/DSS-Induced CAC Mice

**Figure 2:**
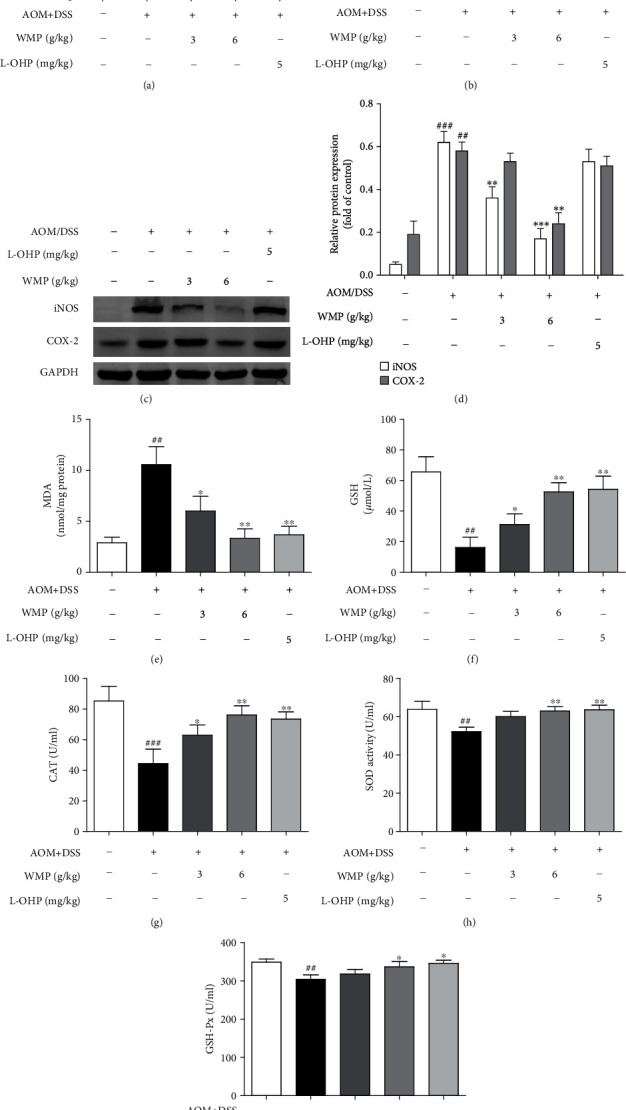
WMP inhibited inflammation and oxidative stress in colon tissues of AOM/DSS-induced CAC mice. (a) ELISA analyses of IL-1*β* level in blood plasma of AOM/DSS-induced CAC mice. (b) ELISA analyses of IL-6 level in blood plasma of AOM/DSS-induced CAC mice. (c) Western blot analyses of the expression of iNOS and COX-2 in the colon tissues of AOM/DSS-induced CAC mice. Representative blots are shown with densitometry (*n* = 3). (d) Quantified western blot results of colon proteins. (e) The MDA level was examined by kit. (f) The GSH level was examined by kit. (G-I) The activities of CAT, SOD and GSH-Px were detected by kits, respectively. Data are expressed as the mean ± SD of 7-10 mice in each group. ^##^*p* < 0.01 and^###^*p* < 0.001 versus the control group; ^∗^*p* < 0.05,  ^∗∗^*p* < 0.01, and^∗∗∗^*p* < 0.001 versus the AOM/DSS group.

Chronic colonic inflammation is a known risk factor for CRC [54]; therefore, the anti-inflammatory effect of WMP on AOM/DSS-induced CAC was herein evaluated. AOM/DSS stimulation notably increased the levels of interleukin-1*β* (IL-1*β*) and IL-6 compared to the control group and administered with WMP restoring the IL-1*β* and IL-6 levels in blood plasma of AOM/DSS-induced CAC mice (Figures 2(a) and 2(b)). L-OHP could also decrease the levels of the above inflammatory factors similar to WMP (Figures 2(a) and 2(b)). The effect of WMP on the protein expression of inflammatory mediators, such as inducible nitric oxide synthase (iNOS) and cyclooxygenase-2 (COX-2), was further determined in the AOM/DSS-induced CAC mice. AOM/DSS stimulation led to the increased expression of iNOS and COX-2, and WMP but not L-OHP decreased the expression of those above proteins (Figures 2(c) and 2(d)). Accumulating evidence has proved that AOM and its metabolite could induce oxidative stress [55]. Therefore, the antioxidative stress effect of WMP on AOM/DSS-induced CAC was further evaluated. Compared to the control group, AOM/DSS stimulation increased malondialdehyde (MDA) level but decreased glutathione (GSH) level in colon tissues of CAC mice. Both WMP and L-OHP restored the abnormal levels of MDA and GSH compared to the AOM/DSS-treated alone group (Figures 2(e) and 2(f)). Catalase (CAT), superoxide dismutase (SOD), and glutathione peroxidase (GSH-Px) are three key antioxidant enzymes catalyzing and negatively regulating oxidative stress [56]. WMP notably restored the decreased enzyme activities of CAT, SOD, and GSH-Px caused by AOM/DSS stimulation in colon tissues of CAC mice, showing strong ability to resist oxidative stress (Figures 2(g)–2(i)). L-OHP also significantly inhibited oxidative stress similar to WMP. Collectively, WMP inhibited inflammation and oxidative stress in colon tissues of AOM/DSS-induced CAC mice.

### 3.3. AHCY Was Highly Expressed in Clinical Samples of Human Colon Cancer and WMP Inhibited the AHCY Expression in Colon Tissues of AOM/DSS-Induced CAC Mice

**Figure 3:**
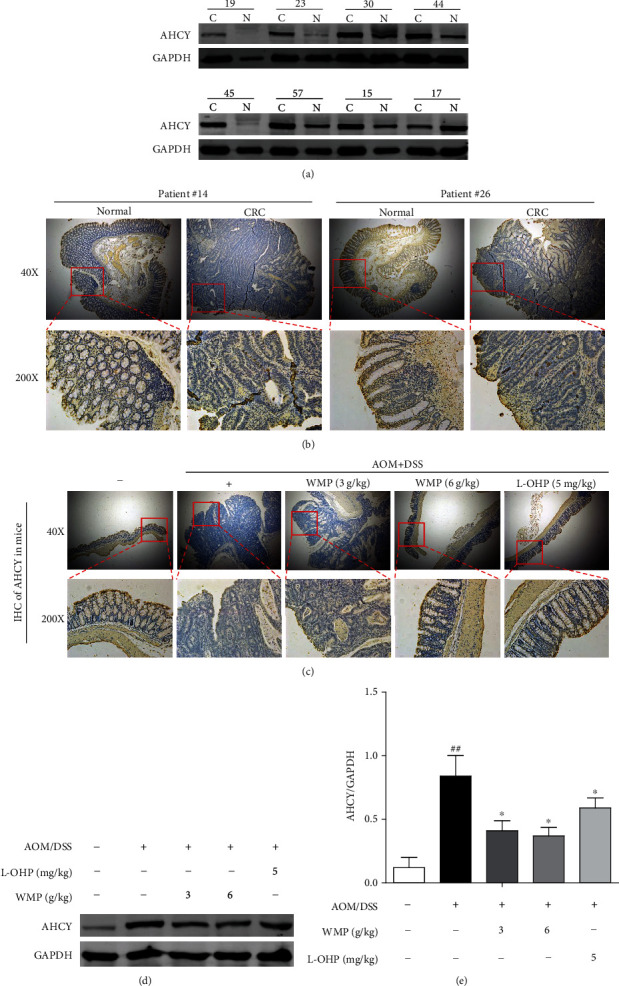
AHCY was high expression in clinical samples of human colon cancer and WMP inhibited the AHCY expression in colon tissues of AOM/DSS-induced CAC mice. (a) Western blot analyses of the expression of AHCY in the colon tissues of AOM/DSS-induced CAC mice. Representative blots are shown with densitometry (*n* = 3). (b) Immunohistochemical analyses of the expression of AHCY in clinical colon cancer samples and the adjacent noncancerous tissues. (c) The effect of WMP on the expression of AHCY was examined by immunohistochemistry. (d) The effect of WMP on the expression of AHCY in colon tissues of AOM/DSS-induced CAC mice was examined by Western blot. Representative blots are shown with densitometry (*n* = 3). (e) Quantitation of the result of western blot. Data are expressed as the mean ± SD (*n* = 3). ^##^*p* < 0.01 versus the control group; ^∗^*p* < 0.05 versus the AOM/DSS group.

Several studies have demonstrated the connections between S-adenosylhomocysteine hydrolase (AHCY) and cancer [57, 58]; however, the roles of AHCY in colon cancer and WMP-induced anti-CAC effect remain to be determined. Therefore, we first examined the AHCY expression in clinical samples of human colorectal cancer. Compared to the adjacent noncancerous tissues, western blot showed that AHCY was highly expressed in clinical samples of human colon cancer (Figure 3(a)). Immunohistochemistry staining also observed the upregulation of AHCY in CRC samples compared to the adjacent noncancerous tissues (Figure 3(b)). Those above data indicated that AHCY participated in the pathogenesis of CRC; however, the roles of AHCY in WMP-induced anti-CAC effect remain to be determined. Therefore, we then studied the effect of WMP on protein expression of AHCY in the AOM/DSS-induced CAC mouse model. As shown by the immunohistochemical and western blot, AOM/DSS stimulation notably increased the AHCY expression; however, treatment with WMP decreased the expression of AHCY (Figures 3(c)–3(e)), highlighting that AHCY involved in the WMP-induced anti-AOM/DSS induced CAC effect in mice.

### 3.4. AHCY Overexpression Induced Cell Proliferation, Colony Formation, Invasion, and Tumor Angiogenesis, Whereas Its Knockdown Impaired Its Oncogenic Function

**Figure 4:**
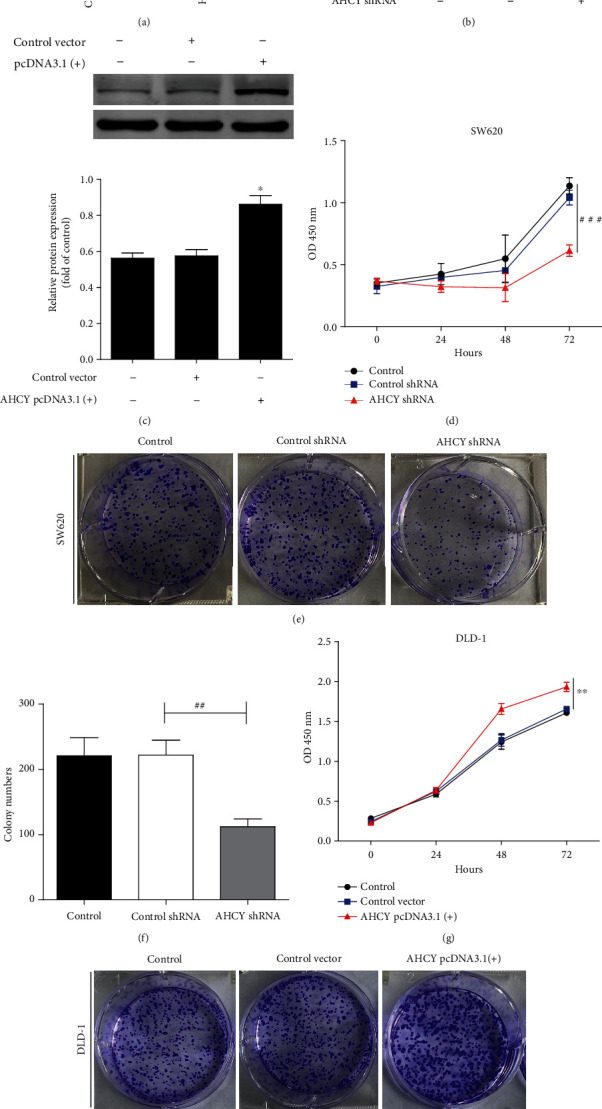
AHCY overexpression induced cell proliferation and colony formation, whereas its knockdown weakened its function. (a) Western blot analyses of the expression of AHCY in nine human colon cancer cell lines SW480, Colo-205, RKO, Caco-2, DLD-1, HCT116, HCT15, SW620, and LOVO. Representative blots are shown with densitometry (*n* = 3). (b) Western blot was used to examine the effect of AHCY shRNA on the expression of AHCY in SW620 cells. (c) Western blot was used to examine the effect of AHCY pcDNA3.1(+) on the expression of AHCY in DLD-1 cells. (d) Effect of AHCY knockdown on tumor proliferation of SW620 cells was examined by CCK8. (e) Effect of AHCY knockdown on colony formation of SW620 cells was examined. (f) Quantitation of the number of colonies. (g) Effect of AHCY overexpression on tumor proliferation of DLD-1 cells was examined by CCK8. (h) Effect of AHCY overexpression on colony formation of DLD-1 cells was examined. (i) Quantitation of the number of colonies. Data are expressed as the mean ± SD (*n* = 3). ^##^*p* < 0.01 and^###^*p* < 0.001 versus the control shRNA group; ^∗^*p* < 0.05 and^∗∗^*p* < 0.01 versus the control vector group.

**Figure 5:**
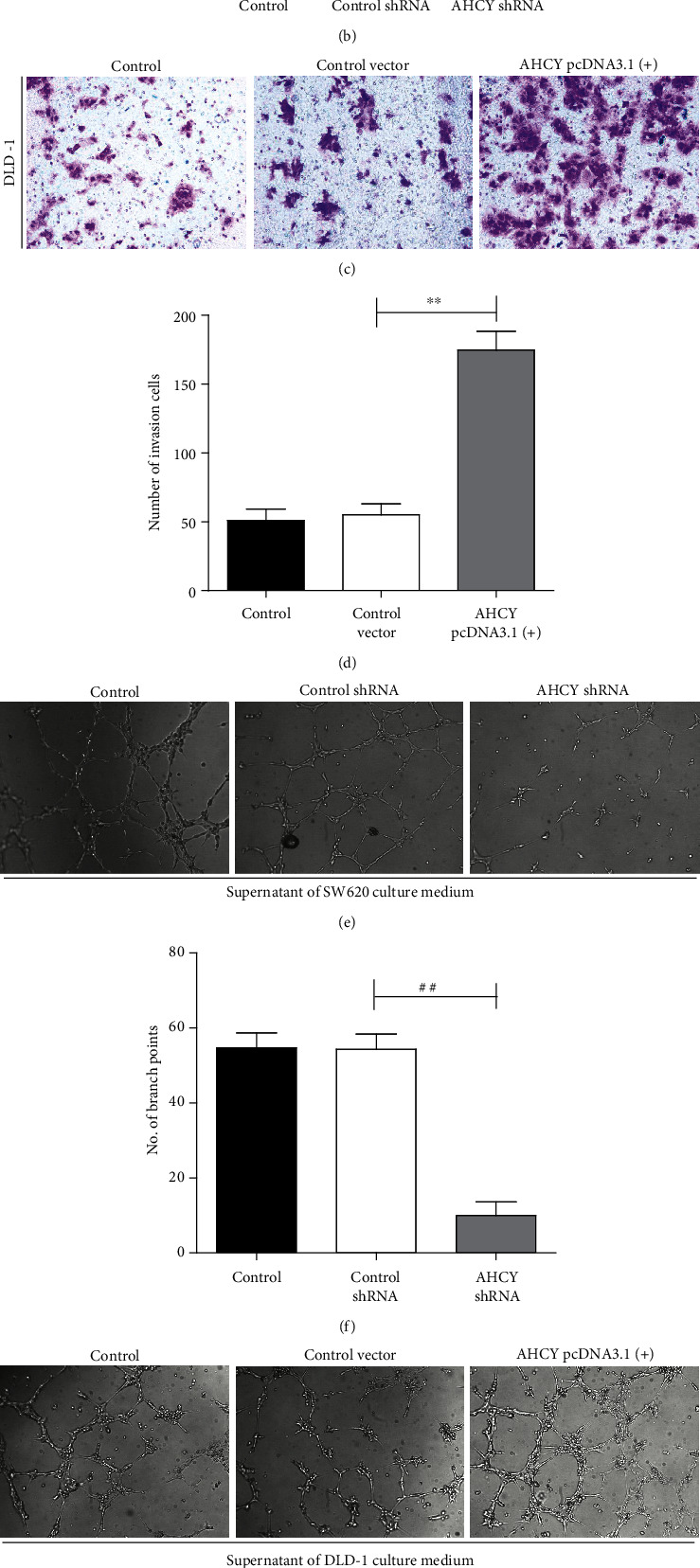
AHCY overexpression induced cell invasion and tumor angiogenesis, whereas its knockdown weakened its function. (a) Effect of AHCY knockdown on tumor invasion of SW620 cells. (b) Quantitation of the number of invasion cells. (c) Effect of AHCY overexpression on tumor invasion of DLD-1 cells. (d) Quantitation of the number of invasion cells. (e) Effect of AHCY knockdown on tube formation of SW620 cells. (f) Quantitation of the number of branch points. (g) Effect of AHCY overexpression on tube formation of DLD-1 cells. (h) Quantitation of the number of branch points. Data are expressed as the mean ± SD (*n* = 3). ^##^*p* < 0.01 versus the control shRNA group; ^∗∗^*p* < 0.01 versus the control vector group.

The above results found that AHCY was upregulated in human colon cancer; however, the regulatory roles of AHCY in colon cancer, especially on tumor proliferation, invasion, and tumor angiogenesis, remain largely unknown. To solve the above question, a total of nine human colon cancer cell lines, i.e., SW480, Colo-205, RKO, Caco-2, DLD-1, HCT116, HCT15, SW620, and LOVO, were used to perform the research. Firstly, the expression of AHCY in nine colon cancer cell lines was examined by western blot. As shown in Figure 4(a), of all the cell lines, SW620 cells had the highest expression of AHCY, whereas DLD-1 showed the lowest expression of AHCY. According to this result, SW620 cells and DLD-1 cells were selected to perform the next study. Secondly, overexpression or knockdown of AHCY by using the related transfection plasmids and transfection efficacy was determined by western blot, respectively. AHCY shRNA plasmid significantly decreased the AHCY expression in SW620 cells, and AHCY pcDNA3.1(+) plasmid evidently increased the AHCY expression in DLD-1 cells, suggesting that the plasmids were successfully constructed (Figures 4(b) and 4(c)). CCK8 assay showed that knockdown of AHCY inhibited the proliferation and colony formation of SW620 cells (Figures 4(d)–4(f)), whereas AHCY overexpression promoted the proliferation and colony formation of DLD-1 cells (Figures 4(g)–4(i)). In addition, knockdown of AHCY also inhibited the invasion of SW620 cells (Figures 5(a) and 5(b)), and AHCY overexpression enhanced the invasion of DLD-1 cells (Figures 5(c) and 5(d)). Moreover, knockdown of AHCY inhibited SW620 cell-induced tumor angiogenesis (Figures 5(e) and 5(f)), while AHCY overexpression promoted DLD-1 cell-induced tumor angiogenesis (Figures 5(g) and 5(h)). Altogether, AHCY overexpression induced cell proliferation, colony formation, invasion, and tumor angiogenesis, whereas its knockdown impaired its oncogenic function.

### 3.5. AHCY Overexpression Enhanced, While Its Knockdown Weakened the Inflammation and Oxidative Stress In Vitro

**Figure 6:**
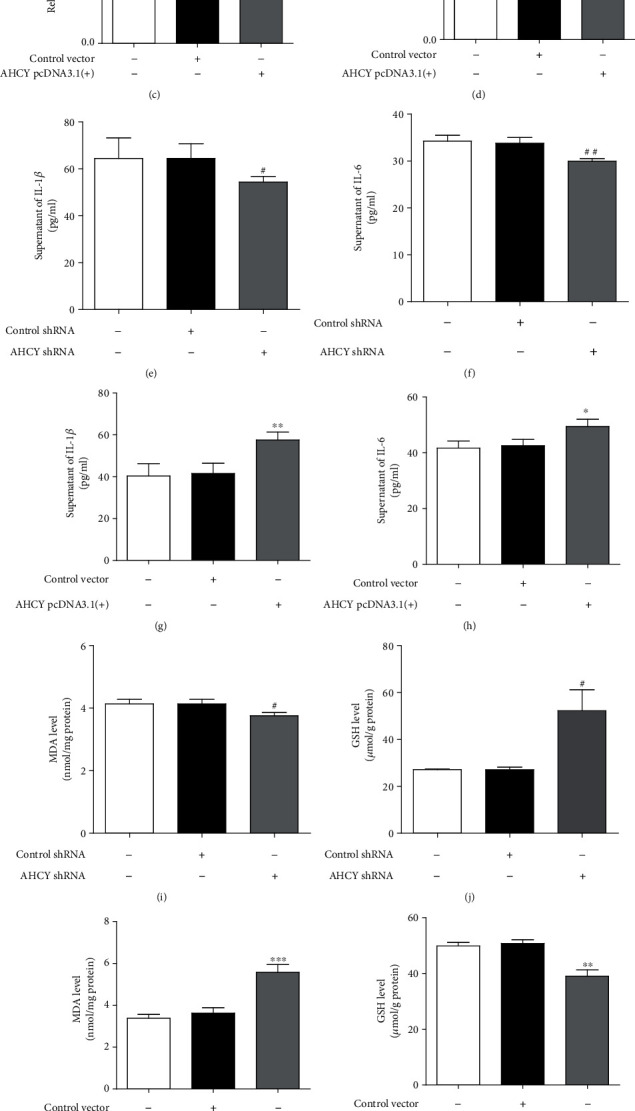
AHCY overexpression enhanced- while its knockdown weakened the inflammation and oxidative stress *in vitro*. (a, b) Western blot was used to examine the effect of AHCY shRNA on the expression of COX-2 and iNOS in SW620 cells, respectively. (c, d) Western blot was used to examine the effect of AHCY overexpression on the expression of COX-2 and iNOS in DLD-1 cells, respectively. (e, f) ELISA was used to examine the effects of AHCY shRNA on the levels of IL-1*β* and IL-6 in the supernatant of SW620 cells, respectively. (G-H) ELISA was used to examine the effects of AHCY overexpression on the levels of IL-1*β* and IL-6 in the supernatant of DLD-1 cells, respectively. (i, j) Effect of AHCY knockdown on the levels of MDA and GSH in SW620 cells, respectively. (k, l) Effect of AHCY overexpression on the levels of MDA and GSH in DLD-1 cells, respectively. (m, n) Effect of AHCY knockdown on the activities of GSH-Px and SOD in SW620 cells, respectively. (o, p) Effect of AHCY overexpression on the activities of GSH-Px and SOD in DLD-1 cells, respectively. Data are expressed as the mean ± SD (*n* = 3). ^##^*p* < 0.01 and^###^*p* < 0.001 versus the control shRNA group; ^∗^*p* < 0.05,  ^∗∗^*p* < 0.01, and^∗∗∗^*p* < 0.001 versus the control vector group.

An *in vivo* study found that WMP inhibited the inflammation and oxidative stress and suppressed the AHCY expression in AOM/DSS-induced CAC mice; however, whether AHCY is involved in the regulation of inflammation and oxidative stress remains to be determined. Thus, we further investigated the regulatory roles of AHCY on inflammation and oxidative stress *in vitro*. Compared to the control shRNA, knockdown of AHCY inhibited the expression of COX-2 and iNOS in SW620 cells (Figures 6(a) and 6(b)), whereas AHCY overexpression increased the expression of the above proteins compared to the control vector in DLD-1 cells (Figures 6(c) and 6(d)). Furthermore, ELISA assay showed that knockdown of AHCY decreased the secretion of IL-1*β* and IL-6 in the supernatant of SW620 cells (Figures 6(e) and 6(f)), and AHCY overexpression increased the secretion of IL-1*β* and IL-6 in the supernatant of DLD-1 cells (Figures 6(g) and 6(h)), highlighting that AHCY positively regulated inflammation in colon cancer cells. Besides, knockdown of AHCY decreased MDA level but increased GSH level in SW620 cells (Figures 6(i) and 6(j)), whereas AHCY overexpression increased MDA level but decreased GSH level in DLD-1 cells (Figures 6(k) and 6(l)). Moreover, depletion of AHCY enhanced the enzyme activities of GSH-Px and SOD in SW620 (Figures 6(m) and 6(n)), while overexpression of AHCY weakened the enzyme activities of GSH-Px and SOD in DLD-1 cells (Figures 6(o) and 6(p)), suggesting that AHCY could promote oxidative stress in colon cancer cells. Taken together, AHCY overexpression was enhanced, while its knockdown weakened the inflammation and oxidative stress *in vitro*.

### 3.6. WMP Suppressed the Hedgehog (Hh) Signaling in Colon Tissues of AOM/DSS-Induced CAC Mice

**Figure 7:**
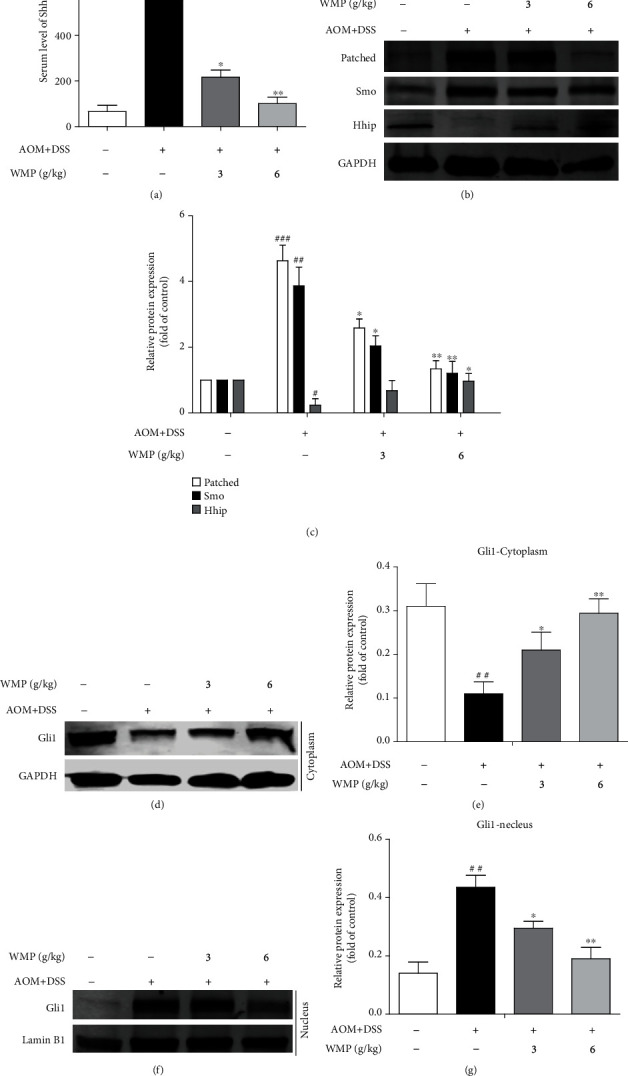
WMP suppressed the hedgehog (Hh) signaling in colon tissues of AOM/DSS-induced CAC mice. (b) Western blot analyses of the expression of Patched, Smo, and Hhip in the colon tissues of AOM/DSS-induced CAC mice. Representative blots are shown with densitometry (*n* = 3). (c) Quantitation of the result of western blot. (d) Western blot analyses of the expression of Gli 1 in the cytoplasm of AOM/DSS-induced CAC mice. Representative blots are shown with densitometry (*n* = 3). (e) Quantified western blot results of colon proteins. (f) Western blot analyses of the expression of Gli 1 in the nucleus of AOM/DSS-induced CAC mice. Representative blots are shown with densitometry (*n* = 3). (g) Quantitation of the result of western blot. Data are expressed as the mean ± SD (*n* = 3). ^##^*p* < 0.01 and^###^*p* < 0.001 versus the control group; ^∗^*p* < 0.05 and^∗∗^*p* < 0.01 versus the AOM/DSS group.

A previous study proved that Hh signaling was involved in the AOM/DSS-induced CAC [37]; however, the effect of WMP on Hh signaling in AOM/DSS-induced CAC remains to be determined. ELISA showed that AOM/DSS stimulation increased the Sonic hedgehog (Shh) level in the blood plasma of mice compared to the control group, and WMP significantly decreased the level of Shh in the blood plasma of mice (Figure 7(a)). Western blot assay found that AOM/DSS stimulation increased the expression of Patched and Smo and decreased the expression of Hhip in the colon tissues of mice; however, they were reversed by treatment with WMP (Figures 7(b) and 7(c)). Moreover, compared to the control group, AOM/DSS stimulation decreased the expression of Gli 1 in cytosolic but increased the expression of Gli 1 in nucleus (Figures 7(d)–7(g)), indicating that AOM/DSS stimulation promoted the nuclear translocation of Gli 1 in colon tissues of mice. Noteworthy, mice were administered with WMP significantly suppressing the nuclear translocation of Gli 1 (Figures 7(d)–7(g)). Altogether, WMP suppressed the Hh signaling in colon tissues of AOM/DSS-induced CAC mice.

### 3.7. AHCY Overexpression Activated the Hh Signaling, Whereas AHCY Knockdown Inactivated the Hh Signaling In Vitro

**Figure 8:**
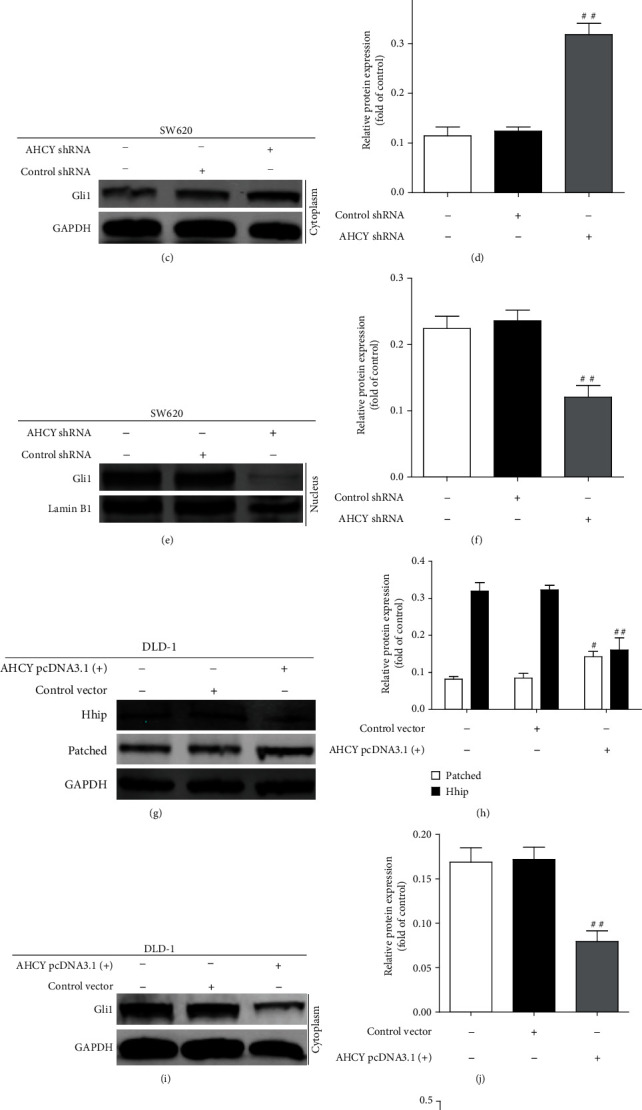
AHCY overexpression activated the Hh signaling whereas AHCY knockdown inactivated the Hh signaling *in vitro*. (a) Western blot was used to examine the effect of AHCY shRNA on the expression of Hhip and Patched in SW620 cells. Representative blots are shown with densitometry (*n* = 3). (b) Quantitation of the result of western blot. (c–f) Western blot was used to examine the effect of AHCY shRNA on the expression of cytoplasm Gli 1 and nucleus Gli 1 in SW620 cells, respectively. Representative blots are shown with densitometry (*n* = 3). (g) Western blot was used to examine the effect of AHCY overexpression on the expression of Hhip and Patched in DLD-1 cells. Representative blots are shown with densitometry (*n* = 3). (h) Quantitation of the result of western blot. (i–l) Western blot was used to examine the effect of AHCY overexpression on the expression of cytoplasm Gli 1 and nucleus Gli 1 in DLD-1 cells, respectively. Representative blots are shown with densitometry (*n* = 3). Data are expressed as the mean ± SD (*n* = 3). ^##^*p* < 0.01 and^###^*p* < 0.001 versus the control shRNA group or control vector group.

The above study demonstrated that WMP inhibited the expression of AHCY and suppressed the Hh signaling in AOM/DSS-induced CAC mice, however, whether AHCY could regulate Hh signaling in colon cancer remain largely unknown. To solve this question, we studied the effects of knockdown or overexpression of AHCY on expression of Hh signaling-associated proteins in colon cancer cells, respectively. Compared to the control shRNA, depletion of AHCY decreased the expression of Patched and increased the Hhip expression (Figures 8(a) and 8(b)) in SW620 cells. In addition, knockdown of AHCY decreased the nuclear expression of Gli 1 but increased the cytosolic expression of Gli 1 (Figures 8(c)–8(f)), suggesting that knockdown of AHCY inactivated the Hh signaling. Besides, compared to the control vector, overexpression of AHCY increased the expression of Patched and decreased the Hhip expression in DLD-1 cells (Figures 8(g) and 8(h)). Moreover, overexpression of AHCY increased the nuclear expression of Gli 1 but decreased the cytosolic expression of Gli 1 in DLD-1 cells (Figures 8(i)–8(l)), highlighting that overexpression of AHCY enhanced the activation of Hh signaling. Collectively, AHCY knockdown inactivated the Hh signaling whereas AHCY overexpression enhanced the activation of Hh signaling in colon cancer cells.

### 3.8. Activation of the Hh Signaling Attenuated the Effects of AHCY Silencing on Inflammation and Oxidative Stress In Vitro

**Figure 9:**
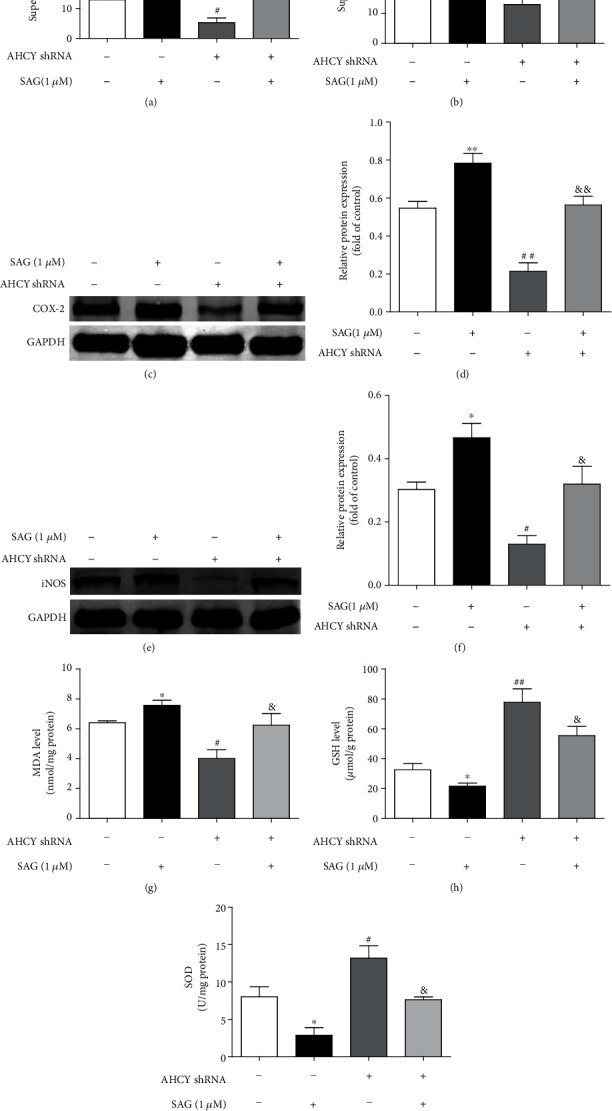
Activation of the Hh signaling attenuated the effects of AHCY silencing on inflammation and oxidative stress *in vitro*. (a, b) Effect of SAG on the levels of IL-1*β* and IL-6 regulated by AHCY knockdown in the supernatant of SW620 cells were examined by ELISA. (c–f) Western blot was used to examine the effect of SAG on the expression of COX-2 and iNOS regulated by AHCY knockdown in SW620 cells, respectively. Representative blots are shown with densitometry (*n* = 3). (g) Effect of SAG on the level of MDA regulated by AHCY knockdown in SW620 cells. (h) Effect of SAG on the level of GSH regulated by AHCY knockdown in SW620 cells. (i) Effect of SAG on the activity of SOD regulated by AHCY knockdown in SW620 cells. Data are expressed as the mean ± SD (*n* = 3). ^∗^*p* < 0.05 and^∗∗^*p* < 0.01 versus the control group; ^#^*p* < 0.05 and^##^*p* < 0.01 versus the control group; ^&^*p* < 0.05 and^&&^*p* < 0.01 versus the AHCY shRNA group.

The above data proved that AHCY played a regulatory role on Hh signaling, and AHCY mediated the regulation of inflammation and oxidative stress in colon cancer cells; however, the roles of Hh signaling in mediating AHCY regulation of inflammation and oxidative stress remain to be determined. To address this scientific problem, we investigated the effect of SAG (Hh signaling agonist) on the inflammation and oxidative stress regulated by AHCY in SW620 cells. Compared to the control group, SAG treatment alone significantly increased the secretion of IL-6 and IL-1*β* together with the increased expression of COX-2 and iNOS (Figures 9(a)–9(f)). Knockdown of AHCY inhibited the secretion of IL-6 and IL-1*β* and decreased the expression of COX-2 and iNOS; however, they were abolished by treatment with SAG in SW620 cells (Figures 9(a)–9(f)). Besides, compared to the control group, SAG treatment alone increased the MDA level but decreased the DSH level and the activity of SOD (Figures 9(g)–9(i)). Depletion of AHCY decreased the MDA level but increased the GSH level and the activity of SOD; however, it was rescued by SAG treatment (Figures 9(g)–9(i)). Taken together, the activation of the Hh signaling attenuated the effects of AHCY silencing on inflammation and oxidative stress in colon cancer cells.

## 4. Discussion

Accumulating evidence has highlighted that inhibition of inflammation and oxidative stress contributed to the control of AOM/DSS-induced CAC [59]. In this study, we found that WMP attenuated the AOM/DSS-induced CAC mainly by suppressing of inflammation and oxidative stress. WMP effectively improved the inflammatory microenvironment around the colon tissues of CAC mice and resisted oxidative stress by enhancing activities of antioxidant enzymes. In particular, AHCY was found involved in the regulation of WMP-induced anti-CAC effect, and WMP could inhibit the AHCY expression in CAC mice. Importantly, we further identified crucial regulatory roles of AHCY in tumor proliferation, colony formation, invasion, and tumor angiogenesis in colon cancer cells. Moreover, AHCY was found participating in the regulation of inflammation and oxidative stress in colon cancer cells. Further mechanism studies found that AHCY promoted inflammation and oxidative stress mainly by enhancing the activation of Hh signaling. To our knowledge, there are few reports that evaluated the role of WMP in the treatment of AOM/DSS-induced CAC by suppressing inflammation and oxidative stress *via* AHCY-mediated Hh signaling pathway.

Several studies have found the connections between AHCY and cancers in recent years. Cao et al. reported that inhibition of DJ-1 potently enhanced the sensitivity of tumor cells to ferroptosis inducers in HT1299 human non-small-cell lung cancer (NSCLC), and the mechanism study found that DJ-1 depletion could inhibit the transsulfuration pathway by disrupting the formation of AHCY tetramer and impairing AHCY activity, suggesting that AHCY linked to the sensitivity of tumor cells to the ferroptosis inducer in NSCLC [60]. Besides, AHCY has been demonstrated as the molecular target of aristeromycin (a derivative of 3-deazaneplanocin A), and treatment of the prostate cancer cells with 3-deazaneplanocin A led to SAH accumulation. Interestingly, knockdown of AHCY by siRNA could also result in the accumulation of SAH and cell growth inhibition of prostate cancer, highlighting that AHCY participated in the regulation of tumor growth of prostate cancer [61]. In addition, a study also proved that AHCY played important roles in hepatocellular carcinoma (HCC), and reduced AHCY activity caused adenosine depletion with activation of the DNA damage response, leading to cell cycle arrest, a decreased proliferation rate, and DNA damage [29], making AHCY a potential target for the treatment of HCC. The present study further proved that AHCY is also involved in the pathogenesis of CRC including AOM/DSS-induced CAC. Moreover, AHCY was found to play essential roles in regulating tumor proliferation, colony formation, invasion, and tumor angiogenesis of colon cancer cells. Therefore, our study further expanded the understanding of the roles of AHCY in cancers. Interestingly, AHCY was also linked to the regulation of inflammation and oxidative stress in colon cancer cells, and AHCY depletion led to the inhibition of inflammation and oxidative stress. Considering that WMP could suppress the inflammation and oxidative stress and inhibited the AHCY expression in CAC mice, we therefore concluded that WMP-induced anti-inflammatory and antioxidant stress effects are, at least partly, through inhibition of AHCY in CAC mice. In the future study, we plan to construct AHCY knockout mice to verify the molecular mechanisms of WMP-induced anti-inflammatory and antioxidative effects *in vivo*.

Hh signaling is a highly conserved evolutionary pathway of signal transmission from the cell membrane to the nucleus [62]. Upon Hh ligand binds to its membrane receptor Patched, the signaling cascade of Hh signaling is initiated [63]. Interestingly, in the absence of Hh, Patched could repress the activity of Smo and inactivate the expression of downstream target genes. However, in the presence of Hh, the inhibitory effect of Patched on the activity of Smo is lifted to trigger the activation of downstream Shh effectors, the glioma-associated (Gli) family of transcription factors [64]. In addition, a study found that the engagement of Hh ligands to Patched can be negatively regulated by endogenous antagonist of Hh ligands, i.e., hedgehog-interacting protein (Hhip). Besides, the translocation of Gli 1 from the cytoplasm to the nucleus is concomitant with the activation of Hh signaling [65, 66]. Hh signaling has been shown to be involved in the AOM/DSS-induced CAC, and suppression of Hh signaling contributed to the amelioration of CAC [67]. Consistent with the previous studies, the present study showed that WMP inhibited the Shh level, decreased the expression of Patched and Smo, and increased the expression of Hhip together with suppression of the nuclear translocation of Gli 1 in AOM/DSS-induced CAC mice, showing potent inhibition effect on Hh signaling in AOM/DSS-induced CAC *in vivo*. Importantly, we identified that AHCY positively regulated Hh signaling in colon cancer cells. Moreover, Hh signaling was found to be involved in the AHCY-mediated regulation of inflammation and oxidative stress in colon cancer cells *in vitro*. Therefore, we further concluded that WMP attenuated AOM/DSS-induced CAC through inhibition of inflammation and oxidative stress by suppressing AHCY-mediated Hh signaling.

There is still a problem to be solved in our future study; that is, how the anti-CAC effect of WMP was linked to AHCY. Though WMP could inhibit the expression of AHCY, however, why the present study focused on the AHCY target should be further elucidated. In fact, our team has been studying the roles of AHCY in colorectal cancer, and we have carried out many aspects of research on AHCY in colon cancer, including the links between AHCY and ferroptosis and the links between AHCY and autophagy. Our team has obtained a large number of unpublished data, and these data remind us that AHCY is a potential and promising target in colon cancer. For the above reasons, in the present study, we tried to investigate whether AHCY was involved in the anti-CAC effect of WMP in mice, and we were pleasantly surprised that WMP could inhibit the expression of AHCY *in vivo*, implying that AHCY participated in the anti-CAC effect of WMP. Therefore, the follow-up in-depth research was also based on such a research finding. As WMP is an herbal formula and includes ten herbs, systematic research is extremely complex. Thus, in the future study, we will use network pharmacology together with the proteomics to further clarify the connection between WMP and AHCY in colon cancer.

In summary, treatment with WMP could effectively alleviate the AOM/DSS-induced CAC. Inhibition of inflammation and oxidative stress by suppressing AHCY may be the relevant mechanisms of WMP for its anti-CAC effect. AHCY is a potential target for the treatment of CAC, and WMP is a promising preventive or protective agent for attenuating CAC.

## Data Availability

The data used to support the findings of this study are available from the corresponding author upon reasonable request.
